# Asymptomatic coronary artery disease in a Norwegian cohort with type 2 diabetes: a prospective angiographic study with intravascular ultrasound evaluation

**DOI:** 10.1186/s12933-019-0832-2

**Published:** 2019-03-09

**Authors:** Satish Arora, Anne Pernille Ofstad, Geir R. Ulimoen, Kåre I. Birkeland, Knut Endresen, Lars Gullestad, Odd Erik Johansen

**Affiliations:** 1 Department of Cardiology, Oslo University Hospital Rikshospitalet, Oslo, Norway; 2 Department of Medical Research, Bærum Hospital Vestre Viken Hospital Trust, Gjettum, PB 800, 3004 Drammen, Norway; 3 Department of Radiology, Akershus University Hospital, Lørenskog, Norway; 4 Department of Transplantation Medicine, Oslo University Hospital Rikshospitalet, Oslo, Norway; 5 Faculty of Medicine, University of Oslo, Oslo, Norway; 6 KG Jebsen Center for Cardiac Research, University of Oslo, and Center for Heart Failure Research, Oslo University Hospital, Oslo, Norway

**Keywords:** Coronary artery disease, Type 2 diabetes mellitus, Invasive coronary angiography, Intravascular ultrasound, Atheroma burden, Multi-factorial treatment

## Abstract

**Aims:**

The prevalence of asymptomatic coronary artery disease (CAD) in type 2 diabetes (T2D) is unclear. We investigated the extent and prevalence of asymptomatic CAD in T2D patients by utilizing invasive coronary angiography (ICA) and intravascular ultrasound (IVUS), and whether CAD progression, evaluated by ICA, could be modulated with a multi-intervention to reduce cardiovascular (CV) risk.

**Methods:**

Fifty-six T2D patients with ≥ 1 additional CV risk factor participated in a 2 year randomized controlled study comparing hospital-based multi-intervention (multi, n = 30) versus standard care (stand, n = 26), with a pre-planned follow-up at year seven. They underwent ICA at baseline and both ICA and IVUS at year seven. ICA was described by conventional CAD severity and extent scores. IVUS was described by maximal intimal thickness (MIT), percent and total atheroma volume and compared with individuals without T2D and CAD (heart transplant donors who had IVUS performed 7–11 weeks post-transplant, n = 147).

**Results:**

Despite CV risk reduction in multi after 2 years intervention, there was no between-group difference in the progression of CAD at year seven. Overall, the prevalence of CAD defined by MIT ≥ 0.5 mm in the T2DM subjects was 84%, and as compared to the non-T2DM controls there was a significantly higher atheroma burden (mean MIT, PAV and TAV in the T2D population were 0.75 ± 0.27 mm, 33.8 ± 9.8% and 277.0 ± 137.3 mm^3^ as compared to 0.41 ± 0.19 mm, 17.8 ± 7.3% and 134.9 ± 100.6 mm^3^ in the reference population).

**Conclusion:**

We demonstrated that a 2 year multi-intervention, despite improvement in CV risk factors, did not influence angiographic progression of CAD. Further, IVUS revealed that the prevalence of asymptomatic CAD in T2D patients is high, suggesting a need for a broader residual CV risk management using alternative approaches.

*Trial registration* Clinical trials.gov id: NCT00133718 (https://clinicaltrials.gov/ct2/show/NCT00133718)

**Electronic supplementary material:**

The online version of this article (10.1186/s12933-019-0832-2) contains supplementary material, which is available to authorized users.

## Introduction

Type 2 diabetes (T2D) is reported to affect 422 million people world-wide, with a projected increase to 642 million by 2040 [1]. Subjects with T2D have at least a two-fold increased risk for cardiovascular (CV) disorders including coronary artery disease (CAD), stroke, peripheral arterial disease, cardiomyopathy and heart failure [2, 3]. Furthermore, prospective trials have identified that the absolute risk of coronary events in patients with T2D is similar to patients with established coronary heart disease without T2D [4, 5]. Although the risk of coronary events can be reduced by aggressive management of co-existing risk factors and prophylactic treatment with aspirin, ACE inhibitor or statins, the universal use of such therapy is debated as the prevalence of asymptomatic CAD in T2D remains unclear with estimates varying between 10 and 60% [6].

Non-invasive screening for asymptomatic CAD in patients with T2D is currently not recommended by the American Diabetes Association [7], predominantly based on the results of the Detection of Ischemia in Asymptomatic Diabetics (DIAD) study [8]. However, according to the European Society of Cardiology, this issue is still under debate and the characteristics of the patients who should be screened for CAD need to be better defined [9].

The accuracy and availability of non-invasive imaging techniques such as CT angiography, including CT angiography derived fractional flow reserve, has improved considerably [10], and these techniques may in many cases be the preferred option due to the associated lower risk of complications than with invasive investigations. However, the utilization of more accurate, invasive techniques, such as coronary angiography and the gold-standard modality of intravascular ultrasound (IVUS) [11] are more appropriate to determine the prevalence of asymptomatic CAD in patients with T2D.

A detailed characteristic of the constitution of the coronary vascular bed could further help refine residual secondary CV risk assessment, e.g., by using maximal intimal thickness (MIT), a predictor of all-cause mortality, myocardial infarction, and angiographic abnormalities [12].

The purpose of the current study was to evaluate the prevalence and extent of asymptomatic CAD in patients with T2D, as compared to a reference population without T2D and without symptomatic CAD, by utilizing coronary angiography and IVUS. The hypothesis was that patients with T2D have a higher silent coronary artery atheroma-burden than those without T2D. Secondly, we explored the effect of a 2-year multi-interventional treatment strategy aimed to reduce CV risk in T2D on progression of angiographic CAD over 7-years of observation. The hypothesis was that reducing CV risk factors is associated with a reduced CAD progression rate.

## Methods

### Patient population

This is the primary IVUS report of the Asker and Baerum Cardiovascular Diabetes (ABCD) study, and a detailed description of this study has been reported previously [13]. In brief, the ABCD study was a prospective, open, randomized, controlled study of 2 years of intensive versus standard care in 120 patients with T2D conducted at the Asker and Baerum Hospital, Gjettum, Norway. Patients were included during the period January 2002 to February 2004. Inclusion criteria were T2D, age 18–75 years, and the presence of one or more additional CV risk factor (defined as hypertension (treated or ambulatory systolic/diastolic blood pressure (BP) ≥ 140/90 mmHg), past or prior smoking, premature CAD in first degree family member (male < 55 years, female < 65 years), microalbuminuria or dyslipidemia (treated or total cholesterol ≥ 5.0 mmol/L, high-density lipoprotein (HDL)-cholesterol (< 1.0 mmol/L in men, or < 1.1 mmol/L in women or triglycerides ≥ 2.0 mmol/L).

Patients were randomized to 2 years of intensive, hospital-based, structured multi-intervention (n = 60) or standard care (n = 60). Structured intensive multi-intervention comprised 6 months of lifestyle intervention (i.e. advice on diet, exercise and smoking cessation and reimbursement of cost associated with training) followed by targeted, pharmacological therapy to reach pre-specified treatment goals (HbA1c ≤ 48 mmol/mol (6.5%), total/LDL cholesterol < 5.0/3.0 mmol/L, systolic/diastolic blood pressure (BP) < 130/80 mmHg). The participants were reviewed every 3 months over a period of 2 years by a diabetologist in the hospital’s out-patient clinic.

The standard care group remained under the care of their general practitioners who were encouraged to continue with treatment according to current (2002) national and American Diabetes Association [14] guidelines (HbA1c < 7%, LDL-cholesterol < 2.6 mmol/L, systolic/diastolic BP < 130/80) and follow-up recommended at 3-monthly intervals.

All participants underwent a comprehensive diagnostic work-up at baseline, including invasive coronary angiography regardless of symptoms or results of the non-invasive cardiac tests. Patients could refrain from invasive testing and still participate in the study, and 91 of the 120 enrolled consented to invasive coronary angiography. At 2 years follow-up, the diagnostic work-up at baseline was repeated, excluding invasive coronary angiography, and all participants were transferred back to the care they had prior to study entry. It was prespecified that the participants should enter a long-term follow-up, and approximately 7 years after inclusion, an additional diagnostic work-up was conducted, including coronary angiography supplemented with IVUS. Of the 120 patients included in the ABCD study, 85 participated in the long-term follow-up [15], and 56 (46.7%) of these patients had coronary angiography performed both at baseline and 7 years. These 56 patients (30 and 26 patients in the multi-intervention and control group, respectively) constitute the population of this sub-study. All 56 patients were free from cardiopulmonary symptoms at baseline.

The reference material consisted of IVUS of donor hearts from 147 non-T2D donors that a priori were free from symptomatic CAD, performed 7–11 weeks post transplantation.

### Angiographic assessment

Coronary angiography was performed with the percutaneous radial or femoral approach using 6F diagnostic catheters (Cordis Corporation, Miami, Fla., USA) and the water-soluble, non-ionic, dimeric contrast medium iodixanol (Visipaque 320 mg/mL; G.E.Healthcare, Oslo, Norway). Coronary artery angiogram data was evaluated by experienced local staff blinded to treatment and was classified as 1-, 2- or 3-vessel disease according to the presence of a stenosis greater than 50% of lumen diameter. Stenosis of the left main coronary artery of > 50% of lumen diameter was considered to be 2-vessel disease. Inter-observer variability of angiographic classifications was 4.9%.

Further quantitative angiographic evaluation was performed using an established scoring system [16]. Coronary segments were graded as grade 0, 1, 2, 3, 4 or occlusion based on the presence of < 25%, < 50%, < 75%, ≥ 75% or occlusion defined as a > 95% diameter stenosis with a severely reduced or no antegrade flow, respectively. CAD severity score was calculated as the average grade of the diseased coronary segments (i.e. ≥ grade 1). CAD extent score was calculated for each patient based on the number of segments exhibiting lesions ≥ grade 1.

### IVUS imaging

The trial protocol specified IVUS examination of the same major epicardial coronary artery (preferentially the left-anterior descending coronary artery) and this was conducted while performing coronary angiography using a 20 MHz, 2.9F, monorail electronic Eagle Eye Gold IVUS catheter (Volcano Corporation Inc, CA, USA). IVUS images were acquired at a rate of 30 frames/s and pullback speed of 0.5 mm/s. Images were stored digitally for off-line analysis conducted after trial closure by a core laboratory (Oslo University Hospital, Rikshospitalet, Oslo, Norway) blinded to patient treatment. IVUS analysis was performed according to the guidelines for acquisition and analysis of IVUS images by the American College of Cardiology and European Society of Cardiology [17]. Contour detection of both the lumen and external elastic membrane (EEM) was performed at approximately 1 mm intervals using validated software (QIVUS, v.3.0, Medis medical imaging systems, Leiden, the Netherlands).

### IVUS endpoints

MIT was utilized as the primary grayscale IVUS efficacy variable. Previous studies have utilized MIT ≥ 0.5 mm as evidence of pathological intimal disease [12] and this cut-off was utilized in the current study. Other secondary IVUS variables were: (i) percent atheroma volume (PAV) which expresses the summation of atheroma areas in proportion to the EEM area using the equation: PAV = ∑ (EEM_area_ − Lumen_area_)/∑EEM_area_) × 100 and (ii) normalized total atheroma volume (TAV) using the equation: TAV = ∑ (EEM_area_ − Lumen_area_)/number of frames) × median number of frames in cohort. IVUS endpoints in the ABCD sub-study population were compared with the non-T2D reference population of heart transplant donors.

### Statistical analysis

Analyses were performed with the SPSS v 24.0 statistical software (SPSS Inc. Chicago, IL). Data is expressed as mean ± SD or as median (interquartile range) as appropriate and a two-tailed p-value < 0.05 was considered statistically significant. Baseline characteristics and IVUS endpoints were compared using Student’s t-test, Mann–Whitney test and Pearson’s Chi squared test as appropriate. Change in angiographic severity and extent score was compared between treatment groups by performing analysis of covariance (ANCOVA) with the baseline value included as a covariate and treatment group as a fixed factor. To account for an age-effect on atheroma-burden, IVUS data was also analyzed with age-stratification (< 50 years, 50–60 years and > 60 years).

## Results

Baseline characteristics of the T2D cohort are given in Table 1. Mean age was 60.0 ± 8.0 years and mean duration of T2D was 5.9 ± 5.7 years. There was no significant difference in baseline characteristics between the treatment groups, and also, the angiographic cohort had a similar baseline characteristic profile as the overall cohort (n = 120) of the ABCD study (data not shown). The reference population’s mean age was 46.0 ± 13.5 years (n = 24 ≥ 60 years).

**Table 1:** Baseline characteristics and treatment allocation of IVUS sub-study population (n = 56)

Demographics	
Patient age (years)	60.0 ± 8.0
Female gender (%)	12 (21)
Duration of T2D (years)	5.9 ± 5.7
Hemodynamics	
Systolic blood pressure (mmHg)	139.4 ± 18.6
Diastolic blood pressure (mmHg)	81.6 ± 9.3
Angiographic findings	
Normal	21 (38%)
Wall changes	17 (30%)
25–50% stenosis	4 (7%)
> 50% stenosis	14 (25%)
Biochemistry	
Hba1c (%)	7.5 ± 1.6
Total cholesterol (mmol/L)	5.0 ± 1.0
Triglycerides (mmol/L)	1.7 ± 1.0
HDL-cholesterol (mmol/L)	1.3 ± 0.4
LDL-cholesterol (mmol/L)	2.9 ± 0.9
Microalbuminuria	26.2 ± 40.8
eGFR (mL/min/1.73 m^2^)(MDRD)	91.8 ± 19.2
hsCRP (mg/l)^a^	0.23 ± 0.34
NT-proBNP (ng/L)^b^	8.6 ± 14.2
Medication	
Any oral antidiabetic medication (%)	42 (75%)
Insulin	8 (14%)
Loop/thiazide diuretic (%)	8 (14%)
ACE inhibitor (%)	9 (16%)
ARB (%)	13 (23%)
Statin therapy (%)	28 (50%)
Acetyl salicylic acid (%)	17 (30%)
Treatment allocated	
Multi-intervention strategy	30 (53%)
Conventional therapy	26 (46%)

*T2D* type 2 diabetes mellitus, *eGFR* estimated glomerular filtration rate, *MDRD* modification of diet in renal disease, *ACE* angiotensin converting enzyme, *ARB* angiotensin receptor blocker

^a^Data for hsCRP (n = 41)

^b^Data for NT-proBNP (n = 34)

## Effects on cardiovascular risk factors

As previously reported [13], following 2 years of intervention there was a significant between-group difference in glycosylated hemoglobin (HbA1c), fasting plasma glucose, blood pressure and lipids favoring the multi-intervention group. Similarly, at the 7 year follow-up, there was a non-significant trend to sustained difference in glycaemia in favor of the multi-intervention group (HbA1c 7.0 ± 1.0% in multi-intervention vs 7.5 ± 1.2% in standard group, p = 0.067, fasting blood glucose 7.4 ± 1.9 mmol/L in multi-intervention vs 9.5 ± 4.2 mmol/L in the standard group, p = 0.03), whereas blood pressure and lipid levels did not differ.

## Angiographic trajectory

The number of patients in the multi-interventional group with 1, 2 and 3-vessel CAD changed from 3 (10.0%), 0 (0%) and 1 (3.3%) at baseline to 4 (13.3%), 2 (6.7%) and 0 (0%) patients at 7 years as compared to a change from 5 (19.2%), 4 (15.4%) and 1 (3.8%) at baseline to 7 (26.9%), 2 (7.7%) and 1 (3.8%) patients at 7 years in the standard group (p = NS).

**Fig. 1:**
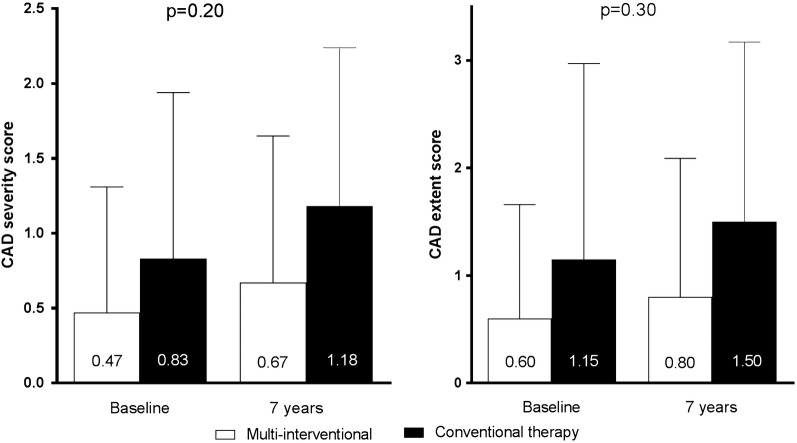
Coronary artery disease (CAD) severity (**a**) and extent (**b**) score according to treatment group, p indicates p-value for between-group difference in change in CAD severity and extent score from baseline to 7 years

CAD severity score increased relatively by 42% (from 0.47 ± 0.84 to 0.67 ± 0.98%) in the multi-interventional group and by 40% (from 0.84 ± 1.11 to 1.18 ± 1.06%) in the standard group from baseline to 7 year follow-up, (p = 0.20 for between-group difference in change, Fig. 1). CAD extent score increased by 33% in the multi-interventional group (from 0.60 ± 1.07 to 0.80 ± 1.30) and by 30% in the standard group (from 1.15 ± 1.83 to 1.50 ± 1.68) from baseline to 7 years (p = 0.30 for between-group difference in change, Fig. 1).

## Grayscale IVUS analysis

**Fig. 2:**
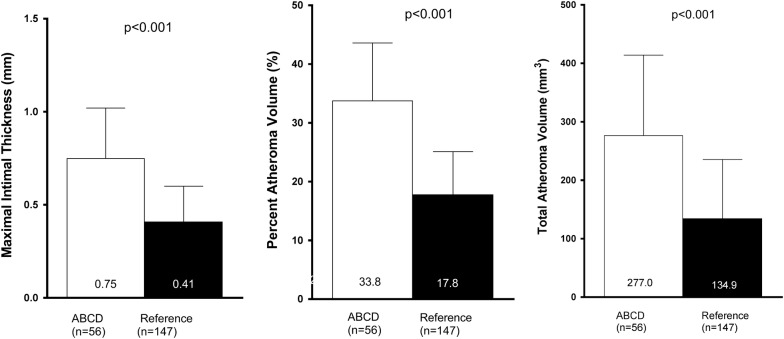
Comparison of quantitative IVUS measurements in the T2D study population (n = 59) with a reference population (donor heart transplants) without known coronary artery disease or type 2 diabetes (n = 147)

**Fig. 3:**
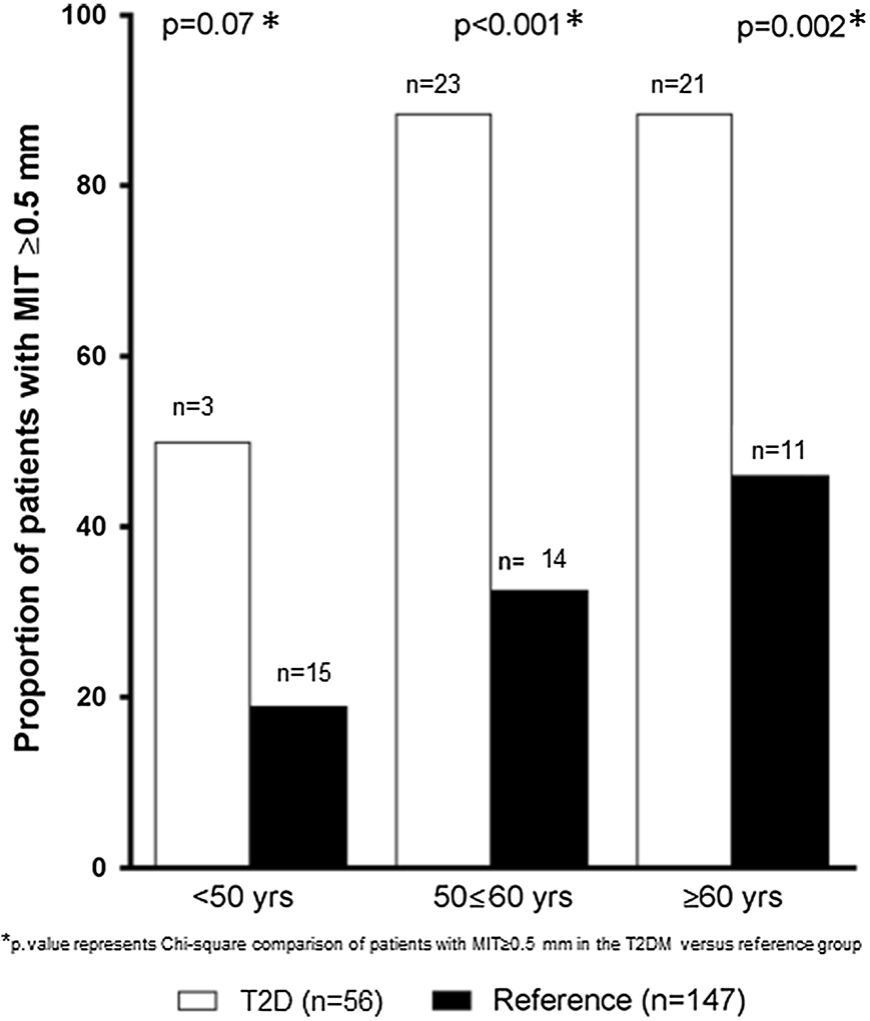
Age-stratified prevalence of coronary heart disease (defined as MIT ≥ 0.5 mm) in the T2D study population (n = 59) with a reference population (heart transplant donors) without coronary artery disease or type 2 diabetes (n = 147). *MIT* maximal intima thickness, *T2D* type 2 diabetes

At the follow-up investigation, the overall mean MIT, PAV and TAV in the T2D population were 0.75 ± 0.27 mm, 33.8 ± 9.8% and 277.0 ± 137.3 mm^3^ as compared 0.41 ± 0.19 mm, 17.8 ± 7.3% and 134.9 ± 100.6 mm^3^ in the reference population (all p-values < 0.05—Table 2 and Fig. 2). Overall, 47 of 56 (83.9%) of the T2D patients had a mean MIT ≥ 0.5 mm as compared to 39 (26.5%) patients in the reference population (p < 0.001). Age-stratified prevalence of CAD (defined as MIT ≥ 0.5 mm) in the T2D population was significantly higher than the reference non-T2D population (p < 0.05, Fig. 3). There was no significant difference between the T2D treatment groups in IVUS parameters with mean MIT, PAV and normalized TAV 0.72 ± 0.26 mm, 32.2 ± 8.6% and 265.1 ± 131.9 mm^3^ in the multi-intervention group as compared to 0.78 ± 0.29 mm, 35.7 ± 10.9% and 290.7 ± 144.5 mm^3^ in the standard group (p-values > 0.05, Table 3 and Additional file 1: Figure S1).

**Table 2:** Comparison of quantitative IVUS results in the ABCD study population (n = 56) with a reference population without T2D and without established CAD (n = 147)

IVUS parameter	ABCD sub-study population (n = 56)	Non-diabetic reference population (heart transplant donors) (n = 147)	p-value
Mean vessel area (mm^2^)	14.7 ± 4.2	16.1 ± 4.0	0.16
Mean lumen area (mm^2^)	9.6 ± 2.8	13.2 ± 3.5	*< 0.001*
Mean plaque area (mm^2^)	5.1 ± 2.5	2.9 ± 1.4	*< 0.001*
Percent atheroma volume (%)	33.8 ± 9.8	17.8 ± 7.3	*< 0.001*
Normalized total atheroma volume (mm^3^)	277.0 ± 137.3	134.90 ± 100.6	*< 0.001*
Mean maximal intimal thickness (mm)	0.75 ± 0.27	0.41 ± 0.19	*< 0.001*
Patients with MIT ≥ 0.5 mm	47 (84%)	39 (26.5%)	*< 0.001*

Italic values indicate significance of p-value (p < 0.05)

*IVUS* intravascular ultrasound, *T2D* type 2 diabetes mellitus, *CAD* coronary artery disease, *MIT* maximal intimal thickness

**Table 3:** Quantitative IVUS analysis of the T2D cohort according to allocated treatment

IVUS parameter	Multi-interventional (n = 30)	Standard therapy (n = 26)	p-value
Mean vessel area (mm2)	14.9 ± 4.3	14.7 ± 4.1	0.86
Mean lumen area (mm2)	10.0 ± 2.9	9.3 ± 2.6	0.37
Mean plaque area (mm2)	4.9 ± 2.5	5.4 ± 2.6	0.49
Percent atheroma volume (%)	32.2 ± 8.6	35.7 ± 11.0	0.19
Normalized total atheroma volume (mm^3^)	265.1 ± 131.9	290.7 ± 144.6	0.49
Mean maximal intimal thickness (mm)	0.72 ± 0.26	0.78 ± 0.29	0.43
Patients with MIT ≥ 0.5 mm	26 (87%)	21 (81%)	0.55

*IVUS* intravascular ultrasound, *T2D* type 2 diabetes mellitus, *MIT* maximal intimal thickness

### IVUS analysis according to baseline coronary angiography

When considering IVUS findings at 7 years according to baseline angiographic findings, there were no significant differences between the treatment groups in MIT or PAV regardless of whether baseline angiography had been normal (n = 21) or shown a stenosis of < 25% (n = 17) or > 25% (n = 18) (Additional file 2: Figure S2). Similarly, there was no significant difference in IVUS parameters between the treatment groups when patients were stratified according to baseline CAD extent score (Additional file 3: Figure S3).

## Discussion

The current ABCD trial is, to our knowledge, the first IVUS investigation of asymptomatic CAD in T2D and demonstrates that the burden of CAD is overwhelmingly high and that CAD progression is substantial. The trial also evaluated the efficacy of a multi-intervention strategy aimed to reduce CV risk, and despite a sustained improvement in glycemic control, such intervention did not influence angiographic progression of CAD.

### Prevalence and detection of coronary artery disease in type 2 diabetes

CAD is the leading cause of morbidity and mortality in patients with T2D. Previously, T2D was considered a CAD equivalent by European and American guidelines [18] implying a high (> 20%) 10-year CV risk for all patients with T2D [18]. However, the validity of this assumption has been questioned in recent years based on data indicating the potentially wide heterogeneity in CV risk among T2D patients [19]. For example, a meta-analysis by Bulugahapitiya et al. included 45,108 patients and revealed a 43% lower risk of developing CAD in patients with T2D without prior myocardial infarction (MI) as compared to patients without T2D with previous MI [20]. Given this heterogeneity, various studies have been performed utilizing methods such as nuclear imaging, echocardiography, carotid ultrasound and exercise stress-testing, to evaluate the impact of non-invasive screening for CAD in asymptomatic patients with T2D [21]. According to these studies, the estimates of CAD prevalence in asymptomatic patients with T2D vary widely with results ranging from 8 to 50% [22]. For example, the DIAD study [8] showed that 22% of patients had abnormal stress myocardial perfusion imaging whereas Rajagopalan et al. [23] found that 58% of asymptomatic patients with T2D patients had abnormal SPECT imaging.

Importantly, surrogate markers of CAD obtained from non-invasive testing carry important prognostic information in asymptomatic individuals with T2D, including coronary artery calcium (CAC) by coronary computed tomography angiography (CTA) [24, 25], carotid atherosclerosis [26] and plaques [25], and aortic stiffness [27]. Despite this and the diverging results of occult CAD prevalence, current guidelines do not recommend universal screening for CAD among patients with T2D, partly based on coronary computed tomography angiography (CTA) studies [28, 29] showing that a sizeable proportion of patients (25–30%) do not have demonstrable plaque on CTA [19]. The ABCD trial is the first trial to date to perform invasive IVUS imaging, a gold-standard technique for evaluation of CAD, in asymptomatic patients with T2D and reveals a CAD prevalence of 84%, indicating a significantly higher burden of disease than previously assumed. Given the increased vulnerability observed in coronary plaques in subjects with diabetes and CAD [30] this prevalence is important to consider. Current guidelines although differentiating their recommendations for choice of glucose lowering medication according to the presence or absence of cardiovascular disease, do however not consider prevalent occult CAD in T2D patients and a refined accurate estimate of risk and disease prevalence is warranted in an effort to improve the applicability and validity of T2D management guidelines.

### Progression of coronary artery disease

A study from 1984 found no significant progression in extent score in a population with CAD of which the majority had their second angiogram performed due to persistent angina [31], but they found that high extent score was an independent, strong predictor of CAD progression. Despite the relatively low extent score in our study, and the high use of statins, we found a substantial progression in both the extent and severity of CAD. This confirms a more aggressive atherosclerosis seen in T2D, and is in line with a Korean study that demonstrated increased progression of coronary artery calcification in those with diabetes as compared to those without [32].

### Multi-interventional therapy in type 2 diabetes

A previous study in patients with T2D and microalbuminuria from the pre-statin era, demonstrated that an intensive multi-interventional therapy program aimed at behavioral modification and pharmacologic therapy targeting hyperglycemia, hypertension, dyslipidemia, and microalbuminuria reduced the risk of CV and microvascular events by about 50 percent [33]. The ABCD trial has previously reported that the 2 year structured, hospital based multi-intervention significantly reduced estimated CV risk in T2D patients [13], however, a subsequent long-term follow-up failed to demonstrate an improvement in CV outcome and mortality [15]. This is congruent with a recent study by Ueki et al. of the Japan Diabetes Outcome Intervention Trial 3 (J-DOIT3) [34], where also a lack of benefit on mortality and CV events was reported despite 8.5 years of effective multi-factorial, target-driven treatment in patients with T2D with additional CV risk factors. The current report of the ABCD trial supports these findings as evidenced by no significant difference in progression of angiographic CAD between the multi-interventional and standard group. Furthermore, there was no significant difference between the multi-interventional and standard group in IVUS parameters 7 years after randomization. However, interpretation of the effect of multi-interventional therapy on CAD assessed by IVUS is limited by the lack of baseline IVUS imaging that does not allow evaluation of disease progression from baseline.

The current neutral results of multi-interventional treatment on CV outcome and CAD extent/severity progression are in concordance with two previous landmark trials. The ACCORD study [35] reported that the use of intensive therapy targeting HbA1c levels did not significantly reduce major cardiovascular events or mortality. The Factor 64 randomized trial [36] utilized coronary CTA to identify CAD in patients with diabetes, and although more aggressive medical therapy in those identified with CAD had a positive effect on lipids, blood pressure, and glucose control, there was no impact on death and coronary heart disease outcomes. Our results conflict however with the DIANA study [37], which showed decreased CAD progression rate with improved glycaemic control after 1 year treatment with voglibose or nateglinide in early T2D. Nevertheless, an intervention period longer than the 2 years in the ABCD trial may ultimately be required to demonstrate a beneficial effect of a multi-interventional strategy on CV outcome and CAD in this population with more advanced T2D and further research is warranted. Furthermore, a more individualized multi-interventional treatment strategy with incorporation of newer therapeutic agents, such as, sodium glucose co-transporter-2 (SGLT-2) inhibitors [38], or glucagon like peptide-1 (GLP-1) agonists [39] with evidence for benefit in patients with CV disease manifestations, may be required to reduce the residual CV risk.

The present study has some limitations. The intervention period was relatively short and the number of patients was relatively small, with also an unintended observation of small baseline imbalances in CAD extent and severity between the treatment groups, which may have influenced the chance to modulate the progression by the intervention. IVUS imaging was not performed at baseline and this limits the possibility for an accurate assessment of CAD disease progression over 7 years in the two treatment groups. Furthermore, the reference population of heart transplant donors is a selected population free from CV risk factors and of notably lower age than the T2D cohort, and may not be representative of the general population. However, the authors believe the data is unique as it provides a gold-standard assessment of CAD prevalence in asymptomatic patients with T2D that challenges the assumptions of current guidelines that are based on non-invasive and less sensitive methods to diagnose CAD.

## Conclusions

In conclusion, this sub-study of the ABCD trial demonstrates that a multi-interventional treatment strategy allows a sustained improvement in glycemic control but does not influence angiographic progression of CAD. IVUS evaluation confirms that an overwhelmingly large proportion of asymptomatic T2D patients have CAD, suggesting that the use of more aggressive and newer prophylactic therapeutic agents addressing residual risk may be warranted.

## Additional files

**Additional file 1: Figure S1.** Maximal intimal thickness (MIT) and Percent Atheroma Volume (PAV) according to treatment group.

**Additional file 2: Figure S2.** Maximal intimal thickness (MIT) and Percent Atheroma volume (PAV) according to treatment group and baseline coronary artery disease (CAD) classified as no disease, angiographic stenosis < 25% and angiographic stenosis ≥ 25%. P for between group difference.

**Additional file 3: Figure S3.** Maximal intimal thickness (MIT) and Percent Atheroma volume (PAV) according to treatment group and baseline coronary artery disease (CAD) extent score. P for between group difference.

## Abbreviations

ABCD: Asker and Baerum Cardiovascular Diabetes study
BP: blood pressure
CAD: coronary artery disease
CTA: computed tomography angiography
CV: cardiovascular
DPP-4: di-peptidyl peptidase-4
EEM: external elastic membrane
GLP-1: glucagon like peptide-1
ICA: invasive coronary angiography
IVUS: intravascular ultrasound
MI: myocardial infarction
MIT: maximal intimal thickness
PAV: percent atheroma volume
SGLT-2: sodium glucose co-transporter-2
TAV: normalized total atheroma volume
T2D: type 2 diabetes

## References

[1] Diabetes Atlas 8th Edition. International Diabetes Federation. www.diabetesatlas.org. Accessed Sept 10 2018.

[2] Stratmann B, Tschoepe D (2011). Heart in diabetes: not only a macrovascular disease. Diabetes Care.

[3] Fox CS (2010). Cardiovascular disease risk factors, type 2 diabetes mellitus, and the Framingham Heart Study. Trends Cardiovasc Med.

[4] Martin-Timon I, Sevillano-Collantes C, Segura-Galindo A, Del Canizo-Gomez FJ (2014). Type 2 diabetes and cardiovascular disease: have all risk factors the same strength?. World J Diabetes..

[5] The American Diabetes Association, The National Heart, Lung, and Blood Institute, The Juvenile Diabetes Foundation International, The National Institute of Diabetes and Digestive and Kidney Diseases, The American Heart Association (1999). Diabetes mellitus: a major risk factor for cardiovascular disease. A joint editorial statement. Circulation.

[6] Bravo PE, Psaty BM, Di Carli MF, Branch KR (2015). Identification of coronary heart disease in asymptomatic individuals with diabetes mellitus: to screen or not to screen. Colombia Med..

[7] Summary of Revisions (2018). Standards of medical care in diabetes-2018. Diabetes Care.

[8] Young LH, Wackers FJ, Chyun DA, Davey JA, Barrett EJ, Taillefer R (2009). Cardiac outcomes after screening for asymptomatic coronary artery disease in patients with type 2 diabetes: the DIAD study: a randomized controlled trial. JAMA.

[9] Ryden L, Grant PJ, Anker SD, Berne C, Cosentino F, Danchin N (2013). ESC Guidelines on diabetes, pre-diabetes, and cardiovascular diseases developed in collaboration with the EASD: the Task Force on diabetes, pre-diabetes, and cardiovascular diseases of the European Society of Cardiology (ESC) and developed in collaboration with the European Association for the Study of Diabetes (EASD). Eur Heart J.

[10] Norgaard BL, Leipsic J, Koo BK, Zarins CK, Jensen JM, Sand NP (2016). Coronary computed tomography angiography derived fractional flow reserve and plaque stress. Curr Cardiovasc Imaging Rep..

[11] Nissen SE, Yock P (2001). Intravascular ultrasound: novel pathophysiological insights and current clinical applications. Circulation.

[12] Tuzcu EM, Kapadia SR, Sachar R, Ziada KM, Crowe TD, Feng J (2005). Intravascular ultrasound evidence of angiographically silent progression in coronary atherosclerosis predicts long-term morbidity and mortality after cardiac transplantation. J Am Coll Cardiol.

[13] Johansen OE, Gullestad L, Blaasaas KG, Orvik E, Birkeland KI (2007). Effects of structured hospital-based care compared with standard care for Type 2 diabetes-The Asker and Baerum Cardiovascular Diabetes Study, a randomized trial. Diabet Med.

[14] American Diabetes Association (2002). Standards of medical care for patients with diabetes mellitus. Diabetes Care..

[15] Ofstad AP, Ulimoen GR, Orvik E, Birkeland KI, Gullestad LL, Fagerland MW (2017). Long-term follow-up of a hospital-based, multi-intervention programme in type 2 diabetes mellitus: impact on cardiovascular events and death. J Int Med Res..

[16] Ledru F, Ducimetiere P, Battaglia S, Courbon D, Beverelli F, Guize L (2001). New diagnostic criteria for diabetes and coronary artery disease: insights from an angiographic study. J Am Coll Cardiol.

[17] Mintz GS, Nissen SE, Anderson WD, Bailey SR, Erbel R, Fitzgerald PJ (2001). American College of Cardiology Clinical Expert Consensus Document on Standards for Acquisition, Measurement and Reporting of Intravascular Ultrasound Studies (IVUS). A report of the American College of Cardiology Task Force on Clinical Expert Consensus Documents. J Am Coll Cardiol.

[18] Bax JJ, Inzucchi SE, Bonow RO, Schuijf JD, Freeman MR, Barrett EJ (2007). Cardiac imaging for risk stratification in diabetes. Diabetes Care.

[19] Budoff MJ, Raggi P, Beller GA, Berman DS, Druz RS, Malik S (2016). Noninvasive cardiovascular risk assessment of the asymptomatic diabetic patient: The Imaging Council of the American College of Cardiology. JACC Cardiovasc Imaging..

[20] Bulugahapitiya U, Siyambalapitiya S, Sithole J, Idris I (2009). Is diabetes a coronary risk equivalent? Systematic review and meta-analysis. Diabet Med..

[21] Rahmani S, Nakanishi R, Budoff MJ (2016). Imaging atherosclerosis in diabetes: current state. Curr Diab Rep..

[22] Bates RE, Omer M, Abdelmoneim SS, Arruda-Olson AM, Scott CG, Bailey KR (2016). Impact of stress testing for coronary artery disease screening in asymptomatic patients with diabetes mellitus: a community-based study in Olmsted County, Minnesota. Mayo Clin Proc..

[23] Rajagopalan N, Miller TD, Hodge DO, Frye RL, Gibbons RJ (2005). Identifying high-risk asymptomatic diabetic patients who are candidates for screening stress single-photon emission computed tomography imaging. J Am Coll Cardiol.

[24] Beller E, Meinel FG, Schoeppe F, Kunz WG, Thierfelder KM, Hausleiter J (2018). Predictive value of coronary computed tomography angiography in asymptomatic individuals with diabetes mellitus: systematic review and meta-analysis. J Cardiovasc Comput Tomogr..

[25] Guaricci AI, Lorenzoni V, Guglielmo M, Mushtaq S, Muscogiuri G, Cademartiri F (2018). Prognostic relevance of subclinical coronary and carotid atherosclerosis in a diabetic and nondiabetic asymptomatic population. Clin Cardiol.

[26] Jeevarethinam A, Venuraju S, Dumo A, Ruano S, Rosenthal M, Nair D (2018). Usefulness of carotid plaques as predictors of obstructive coronary artery disease and cardiovascular events in asymptomatic individuals with diabetes mellitus. Am J Cardiol.

[27] Swoboda PP, Erhayiem B, Kan R, McDiarmid AK, Garg P, Musa TA (2018). Cardiovascular magnetic resonance measures of aortic stiffness in asymptomatic patients with type 2 diabetes: association with glycaemic control and clinical outcomes. Cardiovasc Diabetol..

[28] Raggi P, Shaw LJ, Berman DS, Callister TQ (2004). Prognostic value of coronary artery calcium screening in subjects with and without diabetes. J Am Coll Cardiol.

[29] Anand DV, Lim E, Hopkins D, Corder R, Shaw LJ, Sharp P (2006). Risk stratification in uncomplicated type 2 diabetes: prospective evaluation of the combined use of coronary artery calcium imaging and selective myocardial perfusion scintigraphy. Eur Heart J.

[30] Yoshida N, Yamamoto H, Shinke T, Otake H, Kuroda M, Terashita D (2017). Impact of CD14(++)CD16(+) monocytes on plaque vulnerability in diabetic and non-diabetic patients with asymptomatic coronary artery disease: a cross-sectional study. Cardiovasc Diabetol..

[31] Moise A, Theroux P, Taeymans Y, Waters DD, Lesperance J, Fines P (1984). Clinical and angiographic factors associated with progression of coronary artery disease. J Am Coll Cardiol.

[32] Won KB, Han D, Lee JH, Lee SE, Sung JM, Choi SY (2018). Evaluation of the impact of glycemic status on the progression of coronary artery calcification in asymptomatic individuals. Cardiovasc Diabetol..

[33] Gaede P, Vedel P, Larsen N, Jensen GV, Parving HH, Pedersen O (2003). Multifactorial intervention and cardiovascular disease in patients with type 2 diabetes. N Engl J Med.

[34] Ueki K, Sasako T, Okazaki Y, Kato M, Okahata S, Katsuyama H (2017). Effect of an intensified multifactorial intervention on cardiovascular outcomes and mortality in type 2 diabetes (J-DOIT3): an open-label, randomised controlled trial. Lancet Diabetes Endocrinol..

[35] Gerstein HC, Miller ME, Byington RP, Goff DC, Bigger JT, Buse JB (2008). Effects of intensive glucose lowering in type 2 diabetes. N Engl J Med.

[36] Muhlestein JB, Lappe DL, Lima JA, Rosen BD, May HT, Knight S (2014). Effect of screening for coronary artery disease using CT angiography on mortality and cardiac events in high-risk patients with diabetes: the FACTOR-64 randomized clinical trial. JAMA.

[37] Kataoka Y, Yasuda S, Miyamoto Y, Sase K, Kosuge M, Kimura K (2012). Effects of voglibose and nateglinide on glycemic status and coronary atherosclerosis in early-stage diabetic patients. Circ J.

[38] Zinman B, Wanner C, Lachin JM, Fitchett D, Bluhmki E, Hantel S (2015). Empagliflozin, cardiovascular outcomes, and mortality in type 2 diabetes. N Engl J Med..

[39] Nauck MA, Meier JJ, Cavender MA, Abd El Aziz M, Drucker DJ (2017). Cardiovascular actions and clinical outcomes with glucagon-like peptide-1 receptor agonists and dipeptidyl peptidase-4 inhibitors. Circulation..

