# Supplementary material for: Asymptomatic coronary artery disease in a Norwegian cohort with type 2 diabetes: a prospective angiographic study with intravascular ultrasound evaluation
Source: Cardiovasc Diabetol. 2019 Mar 9;18:26. doi: 10.1186/s12933-019-0832-2 (PMC6408758; doi:10.1186/s12933-019-0832-2)

Supplemental material

Supplementary Figure 1 Maximal intimal thickness (MIT) and Percent Atheroma Volume (PAV) according to treatment group.

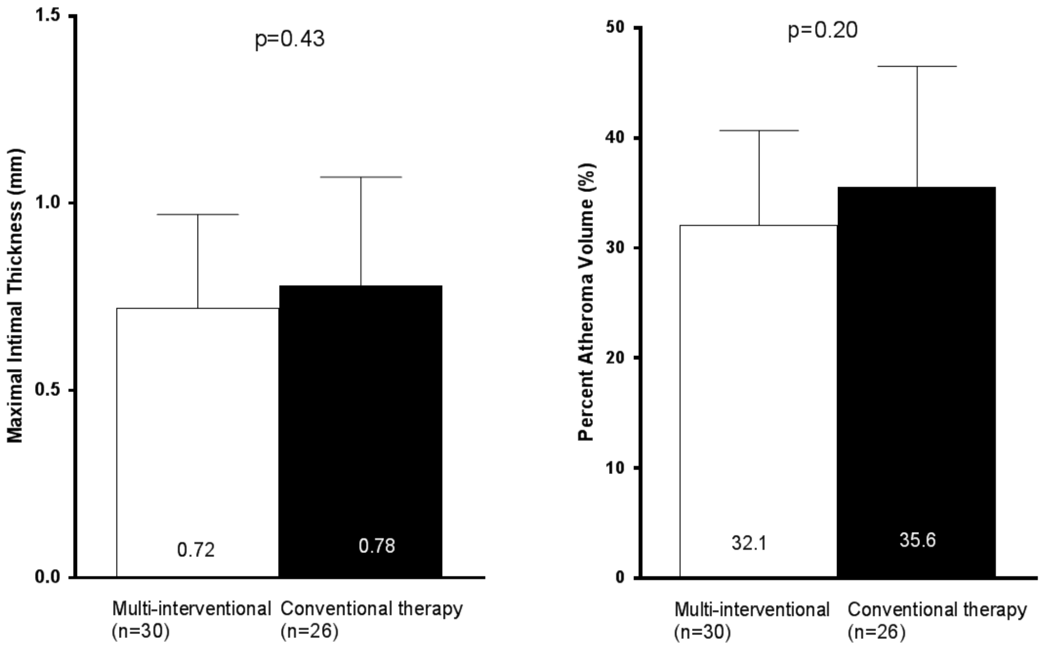

Supplement: Supplementary file 1 — Additional file 1: Figure S1. Maximal intimal thickness (MIT) and Percent Atheroma Volume (PAV) according to treatment group. [file 12933_2019_832_MOESM1_ESM.pdf]
