# Supplementary material for: Asymptomatic coronary artery disease in a Norwegian cohort with type 2 diabetes: a prospective angiographic study with intravascular ultrasound evaluation
Source: Cardiovasc Diabetol. 2019 Mar 9;18:26. doi: 10.1186/s12933-019-0832-2 (PMC6408758; doi:10.1186/s12933-019-0832-2)

Supplementary Figure 2. Maximal intimal thickness (MIT) and Percent Atheroma volume (PAV) according to treatment group and baseline coronary artery disease (CAD) classified as no disease, angiographic stenosis <25% and angiographic stenosis ≥25%. P for between group difference.

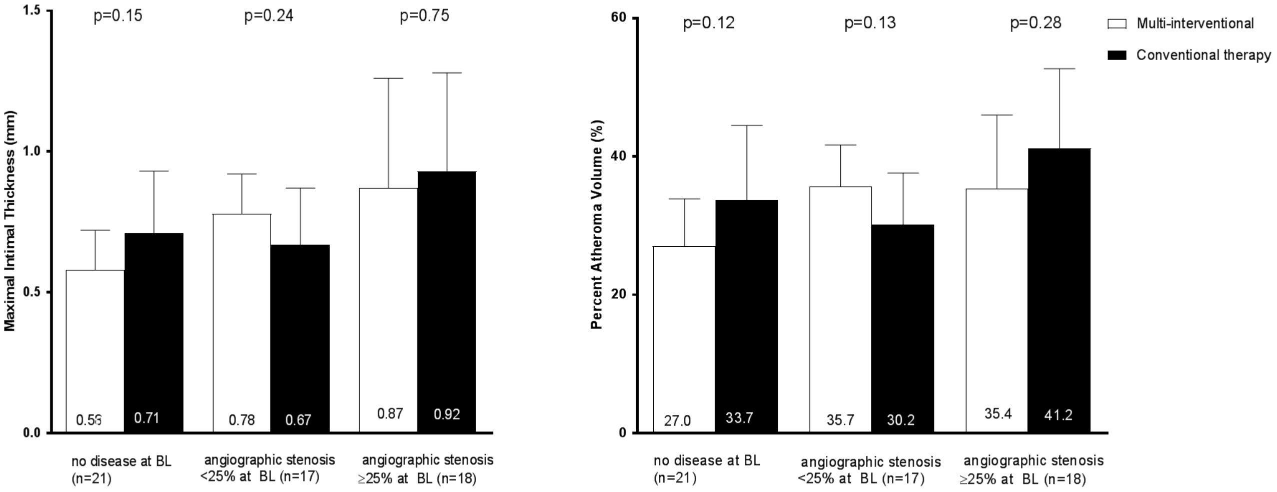

Supplement: Supplementary file 2 — Additional file 2: Figure S2. Maximal intimal thickness (MIT) and Percent Atheroma volume (PAV) according to treatment group and baseline coronary artery disease (CAD) classified as no disease, angiographic stenosis < 25% and angiographic stenosis ≥ 25%. P for between group difference. [file 12933_2019_832_MOESM2_ESM.pdf]
