# Supplementary material for: Asymptomatic coronary artery disease in a Norwegian cohort with type 2 diabetes: a prospective angiographic study with intravascular ultrasound evaluation
Source: Cardiovasc Diabetol. 2019 Mar 9;18:26. doi: 10.1186/s12933-019-0832-2 (PMC6408758; doi:10.1186/s12933-019-0832-2)

Supplementary Figure 3. Maximal intimal thickness (MIT) and Percent Atheroma volume (PAV) according to treatment group and baseline coronary artery disease (CAD) extent score. P for between group difference.

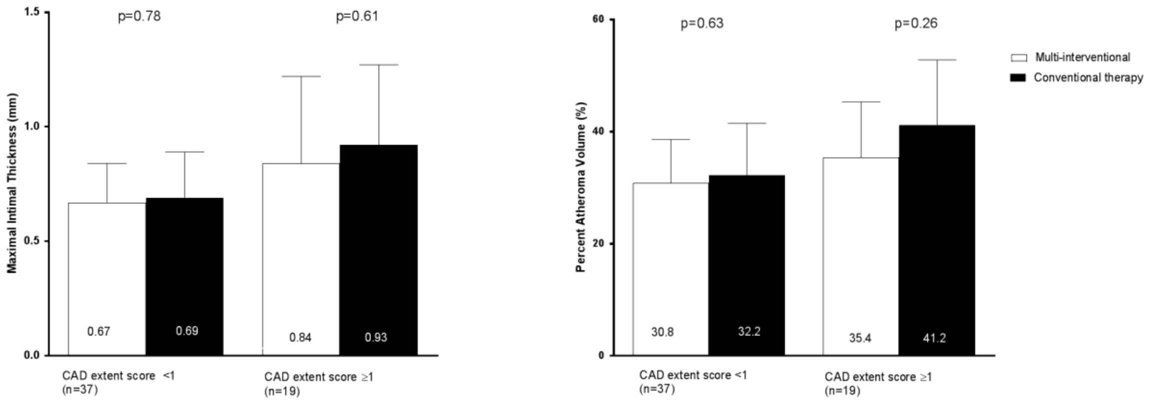

Supplement: Supplementary file 3 — Additional file 3: Figure S3. Maximal intimal thickness (MIT) and Percent Atheroma volume (PAV) according to treatment group and baseline coronary artery disease (CAD) extent score. P for between group difference. [file 12933_2019_832_MOESM3_ESM.pdf]
